# The structure and influencing factors of educationalist spirit among students majoring in education: an integrated model based on entrepreneurial leadership and teacher efficacy

**DOI:** 10.3389/fpsyg.2026.1834394

**Published:** 2026-08-26

**Authors:** Li Jinke, Zhang Siya, Li Yifei

**Affiliations:** 1 Faculty of Education, Zhengzhou Normal University, Zhengzhou, China; 2 School of Economics and Management, Zhengzhou Normal University, Zhengzhou, China; 3 School of Music, Central China Normal University, Wuhan, China

**Keywords:** educational management, educationalist spirit, principal entrepreneurial leadership, teacher efficacy, teacher entrepreneurial leadership

## Abstract

This study aims to explore the internal structure and influencing factors of the educationalist spirit among students majoring in education. The research includes two studies: Study one developed and validated the educationalist spirit scale through exploratory factor analysis and confirmatory factor analysis, which included four dimensions: educational sentiment, innovative spirit, moral leadership, and social responsibility. Based on social cognitive theory, study two constructed an integrated model and examined the joint impact of entrepreneurial leadership and teacher efficacy on the educationalist spirit through a questionnaire survey of 512 university students majoring in education. The results of structural equation modeling analysis indicate that: (1) Entrepreneurial leadership of principal and teacher are significantly positively influence on the educationalist spirit; (2) Principal entrepreneurial leadership has a significant positive impact on teacher entrepreneurial leadership; (3) Teacher efficacy is significantly and positively influence on the educationalist spirit; (4) Teacher efficacy partially mediates the relationship between entrepreneurial leadership and the educationalist spirit. The research conclusion reveals the formation mechanism of the educationalist spirit, providing empirical evidence for normal universities to optimize leadership styles, enhance teacher efficacy, and cultivate future educators.

## 1 Introduction

The construction of a powerful education system relies on basic education as its foundational pillar, is driven by higher education as its propelling force, and is supported by the cultivation of a high-quality, professional teaching workforce as its critical cornerstone (Zhang and Deng, 2025; Zhang and Xu, 2022; Zhao, 2025). Teachers are the primary resource for educational development. In the current era, promoting and cultivating the educationalist spirit has crucial strategic significance. It provides value guidance for the professional growth and professional identity of the teacher group (Yuan, 2025; Hou and Zhang, 2025), on the other hand, it provides practical driving force for deepening education reform and promoting the high-quality development of the education system (John, 1996; Shen and Deng, 2025). As the main educational organizations for cultivating future teachers, normal universities are directly concerned about the prospects and development of the national education in terms of the recognition, internalization, and practice of the educationalist spirit among students majoring in education category. At its core, the educationalist spirit is a spirit of practice, operating through two interrelated mechanisms. Firstly, it encourages teacher to translate their educational philosophies into concrete actions within their future professional work, enabling them to reshape educational realities and advance student development through subjective and proactive engagement. Secondly, it fosters in teacher a resolve to be practice-oriented and strengthens their practical abilities in instruction and education. This is realized concretely by motivating them to discover and resolve problems within educational internships, to reflect and innovate continuously, thus attaining both theoretical refinement and professional maturation through practical immersion (Li and Bai, 2022).

Although the educationalist spirit has become a focal point in policy and theoretical circles, the current literature reveals a critical gap: the existing body of work is predominantly characterized by theoretical conjecture, philosophical deliberation, and policy exegesis, rather than being grounded in empirical evidence (Zhang et al., 2024; Zou and Xie, 2025). The educationalist spirit is essential to today's principals; it is the foundation of education leadership. As the pivotal factor in a school's operation, a principal's spirit defines the institution and exerts a profound influence that extends to teachers, students, and the broader community (Li and Chen, 2025; Lin, 2015). The quality of a principal's leadership is a key determinant of a school's educational excellence and cultural character. Enhancing this leadership relies not merely on skill but fundamentally on the educationalist spirit, which provides its connotation and foundation (Zhang et al., 2024). Particularly for the specific group of education majors, the absence of a reliable, valid, and operable measurement instrument severely impedes the quantification and advancement of research in this field. Zheng and Lu (2024) supported that the cultivation of outstanding normal university students in China currently faces practical challenges such as the homogenization and ambiguity of goal orientation, the disconnection between educational theory and practice, and the closed and rigid nature of teacher training models. This lack prevents an effective assessment of their current level of educationalist spirit, much less the exploration of its underlying mechanisms (Zou and Xie, 2025). Li X. et al. (2025) had highlighted that a distinctive Chinese pedagogical ethos, research on the formation mechanism of the educationalist spirit has predominantly concentrated on theoretical discourse, with relatively limited exploration grounded in the analysis and distillation of biographical accounts and exemplary deeds.

Secondly, there is a lack of a systematic and integrated empirical model in existing research on which factors influence the formation of educationalist spirit and how these factors interact with each other. Some studies may mention the importance of school atmosphere, leadership style, or personal beliefs, while fail to test these variables within a unified theoretical framework (Eesley and Lee, 2021; Li et al., 2024). Recently, entrepreneurial leadership has gained increased recognition in the educational management (Zhu et al., 2023). This concept highlights that teachers funcion not merely as knowledge transmitters but also drivers and innovators of educational transformation. Entrepreneurial leadership has been examined in diverse educational and organizational settings (Eesley and Lee, 2021; He et al., 2024), there are many studies on entrepreneurial leadership and social entrepreneurial leadership conducted with university students (Demir, 2023; Öz, 2022; Öz and Baloğlu, 2024), the link between principal entrepreneurial leadership, teacher behaviors, and organizational effectiveness (Li X. et al., 2025; Liu and Hallinger, 2018). However, despite the growing trend of entrepreneurial leadership research, the intersection between entrepreneurial leadership and the educationalist spirit in relation to students within the Chinese teacher-education context has not drawn attention. While entrepreneurial leadership is conceptually associated with empowerment and proactive value creation, its potential to cultivate the value orientation, sense of mission, and pedagogical commitment of future educators remains largely underexplored (Keyhani and Kim, 2021). Previous research has confirmed the positive correlation between principal leadership and teacher efficacy (Ding and Hong, 2023; Zainal and Matore, 2021), the link between principal entrepreneurial leadership and teacher efficacy (Kalhor et al., 2020; Li X. et al., 2025). Nevertheless, the key driving force of individual behavior, “teacher efficacy”, how to jointly influence the spirit of educators in the interaction with environmental factors is also an unresolved issue.

To fill the research gap mentioned above, this study has established a dual objective: firstly, through study one, to develop and validate a multidimensional scale of educationalist spirit applicable to the students population majoring in education, and provide a reliable measurement tool for it. Secondly, through study two, construct an integrated model based on social cognitive theory, while examining the direct effects of environmental factors (entrepreneurial leadership) and personal cognitive factors (teacher efficacy) on the educationalist spirit, as well as the possible mediating effects of teacher efficacy.

## 2 Literature review

### 2.1 Educationalist spirit and entrepreneurial leadership

The educationalist spirit is a complex concept rooted in the traditional Chinese educational culture and responding to the needs of the times. On the eve of Teacher's Day in 2023, General Secretary Xi Jinping of the People's Republic of China issued documents to outstanding teacher representatives across the country, proposing the unique spirit of educators in China. From six aspects: ideal beliefs, moral sentiments, educational wisdom, practical attitude, benevolence, and pursuit of promoting the Way, the documents deeply expounded its rich connotations and practical requirements, pointing out the direction for the construction of the new era teacher team. It goes beyond basic teaching skills and points toward a deeper and higher professional ideal and realm. Based on existing discussions, it is believed that the educationalist spirit is an organic unity of teachers' profound emotions, outstanding abilities, noble morals, and strong social mission toward the cause of education.

Based on literature analysis, the study preliminary divides its conceptual structure into four core dimensions. Educational passion and care: this refers to a deep love and selfless dedication to the cause of education and students, serving as the source of spiritual motivation. Spirit of innovation and practice: refers to the awareness and ability to break free from conventions and courageously explore innovations in educational concepts, content, and methods (Lan, 2025; Li and Chen, 2025). Moral Leadership and role modeling: it refers to leading by example, guiding students' growth with noble professional ethics and personal charisma, becoming a benchmark for social morality (Xu and Yang, 2024). Social responsibility and mission: it refers to the practice of closely linking one's work to national development and the future of the nation, consciously shouldering the historical responsibility of cultivating talents for the country. Currently, there is an extreme scarcity of empirical research on this construct, and a scientifically valid measurement tool is urgently needed to advance related studies (Li and Chen, 2025; Li X. et al., 2025; Zhang et al., 2024; Zheng and Lu, 2024).

Entrepreneurial leadership initially originated in the business domain, characterized by the ability to foresee and recognize opportunities, take calculated risks, innovate effectively, and inspire team members to work toward a shared vision (Gupta et al., 2004). In recent years, this concept has been introduced to the field of educational management. In educational contexts, entrepreneurial leadership is reflected in school or departmental leaders who identify new opportunities for educational development, foster an organizational climate that encourages innovation and experimentation, empower faculty and students, and unlock their intrinsic potential to collectively pursue the enhancement and transformation of educational quality. Xu and Yang (2024) study suggested that educationalist spirit leads principals to transformational leadership. The principal's role encompasses the dual responsibilities of leading and managing the school. Serving as a key agent in educational reform, an educationalist principal is tasked with actively constructing a new type of school oriented toward the future, a process whose cornerstone is the principal's capacity for leading reform. Zhang et al. (2024) conducted a qualitative analysis of interview data from 32 principals using Nvivo software (Lumivero, Melbourne, Australia) to explore how the educationalist spirit acts upon a main element in shaping the leadership of primary and secondary school principals. Research has found that the educationalist spirit is the embedded core of principal leadership, also deeply shapes the educational philosophy, value orientation, and behavioral patterns of leaders. It is an important driving force for promoting the expansion of leadership from surface to deep, and from theory to practice. Therefore, this research proposes the first hypothesis that:

**H1:** Principal entrepreneurial leadership has a significant positive impact on the educational spirit of students in normal universities.

Entrepreneurial leadership encompasses five dimensions minor behaviors, accelerator behavior, explorer behavior, integrator behavior, and the general entrepreneurial leadership behavior (Thornberry, 2006). Whereas, based on the differences in roles and job functions between principals and teachers, the entrepreneurial leadership is divided into principal entrepreneurial leadership and teacher entrepreneurial leadership. The concept of entrepreneurial leadership in schools is differentiated across organizational tiers: while principals exercise it at the macro institutional level, teachers demonstrate it within middle management roles (Martin et al., 2018). Contemporary models reflect a significant evolution from primarily highlighting the principal's role in implementation and outcomes to recognizing the necessity for teachers to acquire and practice entrepreneurial leadership as a driver of sustainable school development (Hanson, 2017).

Former research has shown that this leadership style can significantly enhance organizational innovation performance and teacher innovation behavior. Li J. et al. (2025) verified that principal entrepreneurial leadership is positively associated with teacher entrepreneurial leadership. Both principal entrepreneurial leadership and teacher entrepreneurial leadership are positively related to teacher efficacy and organizational effectiveness. This study infers that the entrepreneurial leadership perceived by the teachers and their faculties can directly stimulate their own innovative spirit and sense of responsibility by providing a supportive and innovative environment, thereby influencing the formation of their educational spirit. Indeed, interest in the subject also has grown alongside an increase in academic publications focused on entrepreneurship in education. Nevertheless, studies in this field remain at a preliminary stage, and there is still limited understanding of teacher entrepreneurial leadership are and what their professional activities involve (Keyhani and Kim, 2021). Based on the above the research proposes the second and third study hypotheses that:

**H2:** Teacher entrepreneurial leadership has a significant positive impact on the educational spirit of students in normal universities.
**H3:** Principal entrepreneurial leadership has a significant positive impact on teacher entrepreneurial leadership of students in normal universities.

### 2.2 Teacher efficacy and educationalist spirit

Teacher efficacy, derived from Bandura's self-efficacy theory, refers to the belief of teachers in their ability to organize and execute specific teaching tasks, guide students to engage in learning, and effectively address student behavioral issues (Tschannen-Moran and Hoy, 2001). It has been proven to be a key variable for predicting teachers' work engagement, professional commitment, teaching innovation behavior, and career resilience (Hou and Zhang, 2025). Wang et al. (2019) had verified that the effect of spiritual leadership on employee effectiveness from an Intrinsic motivation perspective. According to Zhang and Wang (2024), the educationalist spirit guided the construction of high-quality teacher teams through several key aspects, including conceptual direction, professional ethics, teacher development, innovative pedagogy, team cohesion, curriculum-based ideological education, and research support. The teacher efficacy and the educationalist spirit are interdependent and mutually supportive. The educationalist provides value guidance and internal motivation for teacher efficacy, while teacher efficacy is the concrete manifestation and feedback of the educationalist spirit in practice. Lan (2025) pointed that the educationalist spirit motivates university teachers to proactively engage in professional learning, remain abreast of developments in education and teaching, and ground their practice in students' developmental needs. Through specialized academic training and thematic discussions, they continuously enhance their professional competence, thereby ultimately contributing to the strengthening of teacher collective efficacy. To verify the impact of the educationalist spirit on the collective efficacy of university teachers, Lan (2025) conducted a questionnaire survey and empirical regression analysis on university teachers in Xi'an city. The results showed that university teachers with higher education and professional titles are more able to perceive the connotation and contemporary value of the educationalist spirit, and scored higher in various indicators of teacher collective efficacy.

A strong sense of teacher efficacy fosters enhance the confidence among education majors when navigating anticipated educational complexities. This robust self-assurance subsequently motivates them to pursue more ambitious professional goals and dedicate considerable energy to achieving them. This naturally includes the innovation, dedication, and responsibility required to practice the educationalist spirit. Thereby, this study proposes the fourth hypothesis that:

**H4:** Teacher efficacy has a significant positive impact on the educationalist spirit of students majoring in normal universities.

### 2.3 Entrepreneurial leadership and teacher efficacy

The structured framework of a school creates a hierarchical connection between administrators and teaching staff. This connection, in turn, cultivates constructive relationships and cooperation, both of which are fundamental to achieving organizational effectiveness. The entrepreneurial leadership exhibited by principals acts as a catalyst, significantly shaping teacher behavior by fostering greater motivation, efficacy, innovation, and imagination in their professional practice (Brauckmann-Sajkiewicz and Pashiardis, 2022; Zainal and Matore, 2021). Former research has explored the relationship between principal instructional leadership and collective teacher efficacy (Cansoy et al., 2022), the impact of transformational leadership practices on teachers' self-efficacy (Zainal and Matore, 2021), the influence between entrepreneurial leadership and teaching performance (Kalhor et al., 2020). Recent research investigation were collected 400 questionnaires from teachers in Shandong Province, China. Examined that both principal and teacher entrepreneurial leadership are linked to teacher efficacy as well as organizational effectiveness (Li J. et al., 2025). Based on the previous study, this research proposes the fifth and sixth hypotheses that:

**H5:** Principal entrepreneurial leadership has a significant positive impact on the teacher efficacy of students majoring in normal universities.
**H6:** Teacher entrepreneurial leadership has relationship with the teacher efficacy of students majoring in normal universities.

### 2.4 Entrepreneurial leadership, teacher efficacy and educationalist spirit

The influence of principal entrepreneurial leadership operates primarily through pathways of structural empowerment and sensemaking. By providing institutional support, allocating key resources, and fostering an entrepreneurial culture, it directly empowers teachers to engage in pedagogical innovation (teacher entrepreneurial leadership) and profoundly reinforces their professional calling and endogenous drive (educationalist spirit). Teacher efficacy and teacher entrepreneurial leadership form a mutually reinforcing relationship and jointly enhance the educationalist spirit. Furthermore, teacher efficacy plays a key mediating role in translating external leadership support and intrinsic spiritual motivation into tangible educational innovation practices.

Across the continuum of teacher development in Gansu Province, from pre-service training to in-service programs, a core objective is to guide educators in redefining their professional role. The goal is to transition from being knowledge transmitters to learning collaborators, guides, and caring facilitators, with a focus on fostering students' holistic development—attending to their intellectual, emotional, and social growth—and respecting their individual differences (Lei, 2024). Gansu Province is committed to fostering a competent and professional teaching workforce, continuously enriching its educators' pedagogical expertise. This endeavor aims to ensure that every student has the opportunity to excel and to cultivate a new generation capable of undertaking the mission of national rejuvenation. This measure further demonstrates the importance and mutual influence between developing leadership, teacher efficacy and educationalist spirit. Zheng and Lu (2024) stressed that with the continuous deepening of education reform and the promotion of the Excellent Teacher Training Program, there is still lack clear and definite conclusion on the training objectives, content, and mode of teacher training, specifically, how to lead the outstanding growth of teacher with the educationalist spirit and innovative leadership. As a result, the process of cultivating outstanding teacher trainees presents a real problem of discontinuity, manifested in the detachment between goals and actions, the disconnection between theory and practice, and the closed and rigid training mode (Zheng and Lu, 2024).

The pathway to cultivating the educationalist spirit involves establishing institutional mechanisms at the national level to stimulate its emergence, fostering a supportive cultural atmosphere at the societal level, and forging a core internal drive at the individual level (Li X. et al., 2025). Meanwhile, according to the tripartite interactive determinism of environment individual behavior in social cognitive theory, environmental factors (such as leadership) indirectly influence behavior by influencing individual cognitive factors (such as self-efficacy). An environment full of entrepreneurial leadership can effectively enhance students' ability beliefs and improve their sense of teacher efficacy as well as educationalist spirit by providing resource support, successful role models, and positive feedback. Therefore, this study further proposes the seventh hypothesis that:

**H7:** Teacher efficacy plays a mediating role in the relationship between entrepreneurial leadership and educationalist spirit.

### 2.5 Theoretical framework and hypotheses summary

**Figure 1:**
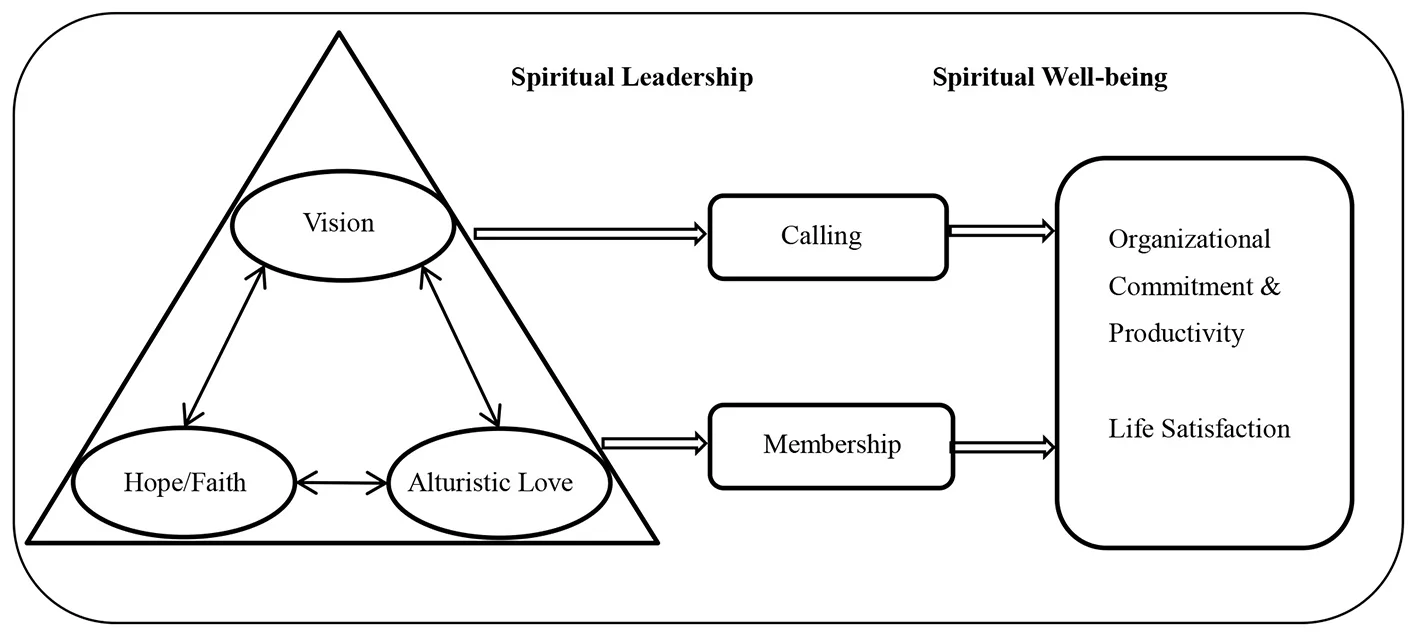
Spiritual leadership theoretical model.

Spiritual leadership theory was developed within the intrinsic motivation model (Fry, 2003; Wang et al., 2019). Fry (2003) and Fry et al. (2011) define spiritual leadership as the values, attitudes, and behaviors required to intrinsically motivate oneself and others to have a sense of spiritual wellbeing. Practicing inner life/mindfulness as a source of hope/faith, vision, and altruistic love that promotes a sense of spiritual wellbeing through calling and membership will affect individual and organizational outcomes (Fry et al., 2016; Subhaktiyasa et al., 2023). Figure 1 showed Fry's spiritual leadership theoretical model.

**Figure 2:**
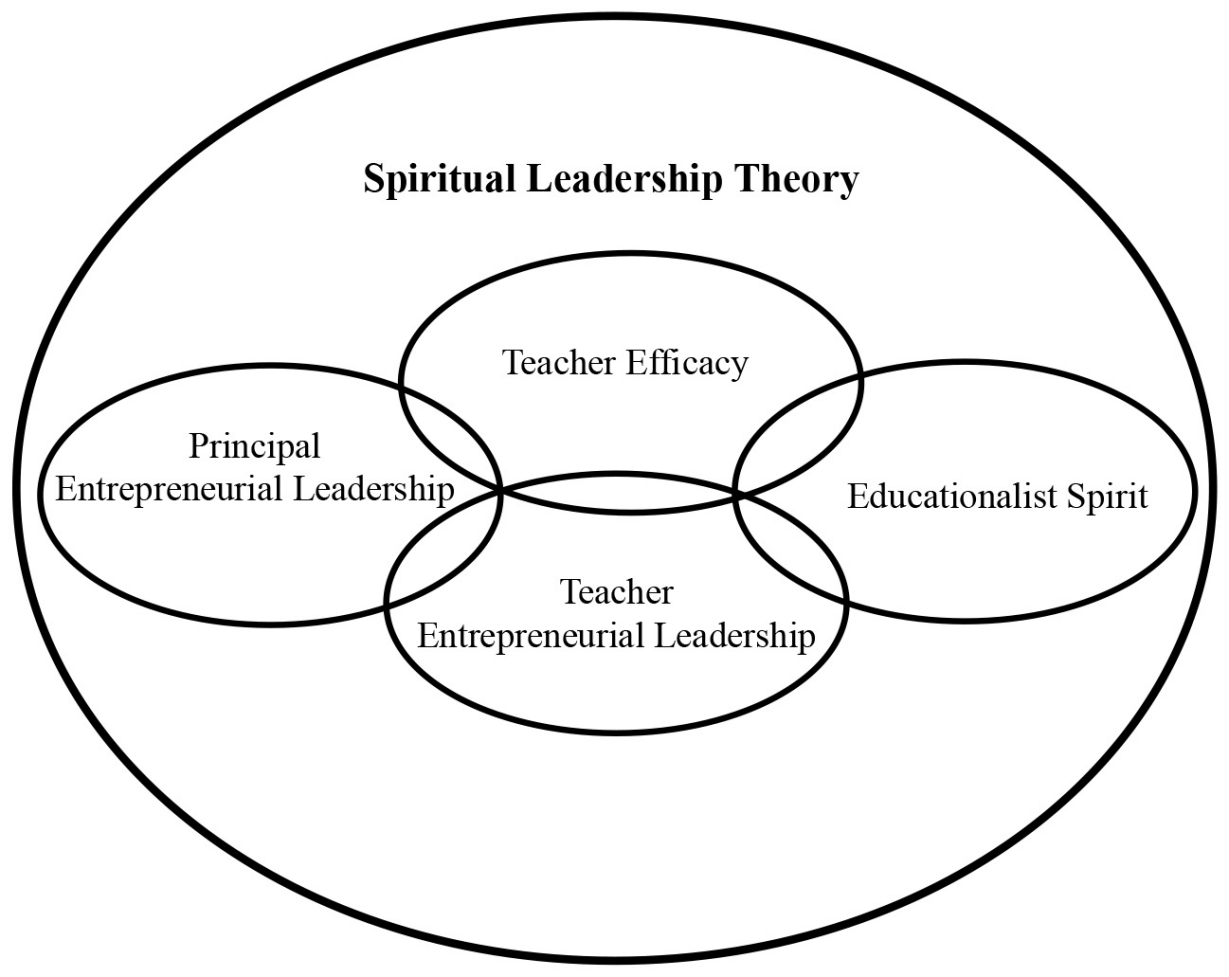
The theoretical framework.

Spiritual leadership, as a fundamental driving force, permeates the entire theoretical framework. It positions at the core, serves as the source and foundation of all energy within the system. It radiates inward, gradually permeating and nourishing variables across all peripheral levels, thereby constructing the essential theoretical construction for its operation. The four variables in the inner layer—principal entrepreneurial leadership, teacher entrepreneurial leadership, teacher efficacy, and the educationalist spirit—function not in a linear causal sequence, while through dynamic reciprocity and mutual reinforcement within the field of spiritual leadership theory. Synthesizing the preceding literature review, this study proposes the theoretical framework presented in Figure 2. The framework posits both a direct effect of entrepreneurial leadership on the educationalist spirit and an indirect effect mediated through the enhancement of teacher efficacy.

**Figure 3:**
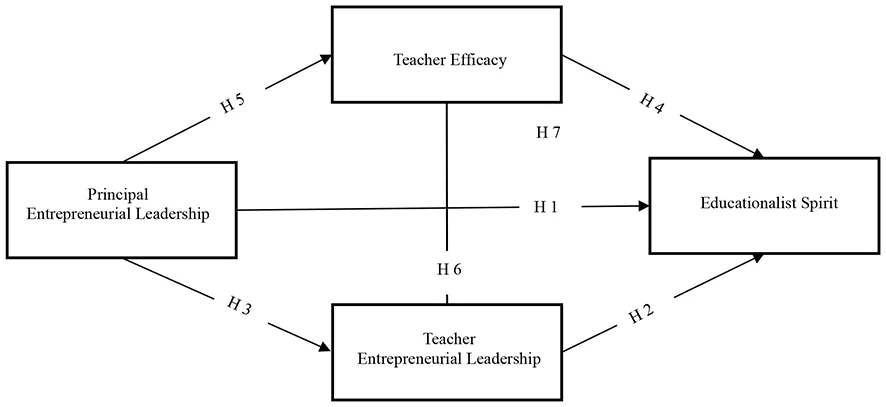
Research model.

This study has significant theoretical and practical implications. Theoretically, it serves to operationalize and quantify the core construct of the educationalist spirit, which enabling a deeper examination of its formative mechanisms through the development and validation of a dual-pathway model. The findings of this research could further contribute to acknowledging and supporting existing principal and teacher entrepreneurial leadership, while also helping to create conditions that allow new entrepreneurs in education to thrive. In practice, research conclusions can provide clear guidance for managers and teachers of normal universities to cultivate future educators accurately and efficiently by consciously shaping an entrepreneurial leadership environment and systematically enhancing the teaching effectiveness of education majors. Figure 3 is the diagram of proposed research model in this study.

## 3 Methodology

### 3.1 Research design and participants

The total land area of China is about 9.6 million square kilometers, with 34 provincial-level administrative regions (China Government Network, 2025). There are a total of 13 normal universities in Henan Province. In order to make the data sample more representative, Henan Normal University (the best normal university in Henan Province for cultivating educational talents nationwide) and Zhengzhou Normal University (a general normal university dedicated to serving local primary and secondary schools and training future teachers and principals) were selected for research. It should be noted that in this study, in the Chinese higher education system, the term “students major in education” in normal universities specifically refers to applied talents who are registered in the Teacher Education Program, with primary and secondary school or kindergarten teachers as their first career goal, and receive systematic teaching skills training. This group has a clear career orientation, their training program is guided by national or local education administrative departments, and the vast majority of students will obtain the corresponding subject teacher qualification certificate upon graduation. Therefore, the students major in education in this study have a dual attribute. They possess both theoretical literacy in the field of education and the professional mission to undertake future educational and teaching practices. This specific identity trait makes it of unique practical significance to explore the structure and influencing factors of its educationalist spirit, entrepreneurial leadership, and teacher efficacy.

This study adopted a cross-sectional survey design and included two sub studies. The research subjects were full-time students from two normal universities (Henan Normal University and Zhengzhou Normal University) in Henan Province, China. Using random sampling method, questionnaires were distributed through online questionnaire platforms (Questionnaire Star App) and offline face-to-face channels in the targeted univeristies. A total of 587 questionnaires were collected and permission consent obtained from individual participant, and after excluding invalid questionnaires with short response time and regular responses, 512 valid questionnaires were obtained. The effective response rate was 87.2%. In the sample, males accounted for 21.5% and females accounted for 78.5%; Freshman to senior students accounted for 25.2%, 28.9%, 36.6%, and 9.3% respectively; The secondary subjects of students mainly included elementary education faculty, educational science faculty, and special education faculty.

### 3.2 Instruments

The questionnaire consisted of 93 items, including two parts A and B. Part A had three demographic information items: gender, age, and study majors. Part B was the 5-point Likert scale, it contained 16 items on educationalist spirit, 50 items on principal entrepreneurial leadership and teacher entrepreneurial leadership, 24 items on teacher efficacy.

Educationalist spirit scale. A multi-phase development procedure was employed to create this scale. First, initial item generation: an initial item pool of 20 was created based on a synthesis of the relevant literature and in-depth interviews with four domain experts (Expert A: University Putra Malaysia, faculty of educational studies, associate dean and professor, doctoral supervisor, with a research focus on educational leadership and sustainable development in education. Expert B: professor and vice dean of the teacher education research institute, Central China Normal University, with a research expertise in teacher professional development and professional identity. Expert C: professor and doctoral supervisor at the department of education, Henan Normal University, specializing in the development of teacher ethics and educator spirit. Expert D: postdoctoral fellow of the department of education at Zhengzhou Normal University, specializing in the fields of digital education, higher education management, and educational psychology). Then, content validity assessment: the initial items were evaluated by an independent panel of the education specialists. The specific participation process of the four experts is as follows, referring to the scale development guidelines of Lynn (1986), four experts independently read the 20 preliminary items of the educationalist spirit, and based on Lynn's (1986) content validity evaluation criteria, rated the relevance, clarity, and representativeness of the initial 20 items, then discussed the items with differences one by one through online meetings. Finally, merge 3 items into 2 items, modify 2 wording items, and delete 3 items to form the initial version of the scale with 16 items. Third, psychometric properties: though exploratory factor analysis, the finalized 16-item scale exhibited satisfactory reliability and validity in this study. As future teachers, the educationist spirit of students is a key psychological trait that influences their professional identity and future career development, which measures students' degree of identification and internalization of the four dimensions contained in the educator spirit, from patriotism to educational mission. Additionally, the study introduces entrepreneurial leadership as well as teacher efficacy as antecedent variables, aiming to explore how these factors work together to form the educationalist spirit.

Entrepreneurial leadership scale utilized the Thornberry (2006) entrepreneurial leadership questionnaire with five dimensions for measuring the entrepreneurial leadership performance of principal and teacher from the perspective of students in normal universities. It measures whether students possess traits such as vision building, risk-taking, and proactivity when facing educational innovation and challenges. Meanwhile, students' perceived entrepreneurial leadership of their principals behavior is evaluated. This part is measuring the leadership atmosphere in the educational field where the students are located. As future teachers, students are learners, also observers and experiencers of school culture. Their perception of the principal's management style directly affects their cognitive schema of the future career environment, which is precisely the key entry point for the study to explore the external factors influencing the educationalist spirit. In addition, teacher efficacy scale adopted the Tschannen-Moran and Hoy (2001) teachers' sense of efficacy questionnaires with three dimensions. The scale had been validated by multiple scholars and had good reliability and validity. Measure the self-efficacy beliefs of students toward their future teaching abilities. This scale includes three dimensions: instructional strategies effectiveness, classroom management effectiveness, and student engagement effectiveness. The scale measures students' confidence in effectively organizing teaching and stimulating student motivation when they pursue a teaching career in the future, which aims to measure their internal psychological resources as prospective teachers.

### 3.3 The analytical approach

The data collection of the universities was carried out through a questionnaire distribution online platform and face-to-face offline channels. After obtaining informed consent from the schools and students, the questionnaire links were pushed through class groups. The data collection period took 6 weeks. The data analysis was conducted using SPSS 25.0 and AMOS 24.0 software (IBM Corp., Chicago, IL, USA), with the following steps: Study 1: randomly divided the total sample into two parts (256 each). Conducting exploratory factor analysis on sample A and confirmatory factor analysis on sample B to test the construct validity, reliability, and criterion related validity of the educationalist spirit scale. Study 2: referring to all samples (*N* = 512), firstly, use SPSS for common method bias testing, descriptive statistics, and correlation analysis for assessing the overall description and correlation testing of the database. Subsequently, structural equation modeling was used to test hypotheses H1, H2, H3, H4 and H5. Finally, Bootstrap method (repeated sampling 5,000 times) was used to test the mediating effect H7.

## 4 Findings

### 4.1 Development and validation of the instruments

To determine the optimal number of factors for the educationalist spirit Scale, the study first conducted parallel analysis (Horn, 1965) prior to exploratory factor analysis. This approach compares eigenvalues derived from the actual data against those generated from random data via Monte Carlo simulation (1,000 permutations). Factors with eigenvalues exceeding the corresponding 95th percentile random thresholds are retained (Hayton et al., 2004). The first four eigenvalues from the actual data (5.84, 2.96, 2.03, 1.47) all exceeded their corresponding 95th percentile thresholds from random data (1.62, 1.55, 1.48, 1.42), whereas the fifth eigenvalue (1.05) fell below its threshold (1.38). Accordingly, a four-factor solution was retained.

Next, to verify the scientific validity of the dimension structure of the educationlist spirit scale and ensure the validity of subsequent hypothesis testing, this study employed cross validation (involving both exploratory factor analysis and confirmatory factor analysis) (Anderson and Gerbing, 1988; Fidell, 2012). Firstly, the total sample (*N* = 512) was randomly divided into two independent sub samples using SPSS. Exploratory factor analysis was conducted to determine an underlying construct of the items, and Confirmatory factor analysiswas used to assess the fit of this underlying construct to observed data. Firstly, exploratory factor analysis was conducted on sample A (*n* = 256). The KMO value is 0.685, and the Bartlett sphericity test is significant (χ^2^ = 318.650, *p* < 0.001), indicating that the data is suitable for factor analysis. Using principal component analysis and maximum variance rotation, four factors with eigenvalues greater than 1 are extracted, and the cumulative explained variance variation is 68.42%. The factor loading of each question item are above 0.65, and there is no cross loading, indicating a clear factor structure. Although the four factors in this study were theoretically expected to be somewhat correlated, we chose Varimax orthogonal rotation at the exploratory stage to obtain a parsimonious and independent factor structure, which would allow for a more rigorous test of discriminant validity via confirmatory factor analysis in the subsequent step (Fabrigar et al., 1999). The subsequent CFA results confirmed the robust discriminant validity of the four-factor model, supporting the appropriateness of orthogonal rotation in this study. Then, conduct reliability analysis on the scale. The consistency level of the 16 items in the scale reached 0.869, which is higher than the standard of 0.7. Each dimension (1–5: 0.857; 6–9: 0.764; 10–13: 0.755; 14–16: 0.787) is higher than 0.7, indicating good reliability of the scale. Second, confirmatory factor analysis was conducted on sample B (*n* = 256). The four-factor model demonstrated acceptable fit indices: χ^2^/df = 2.15, CFI = 0.96, TLI = 0.95, RMSEA = 0.067, SRMR = 0.041. All metrics met acceptable standards, indicating that the four-factor structure was well-supported by the data. The composite reliability of the scale was 0.92, and the average variance extracted for all four dimensions exceeded 0.50, indicating that the scale possesses good reliability and convergent validity.

Due to CFA's confirmation of the robustness of the scale's dimensional structure and the parameter stability of the measurement model across all data, in order to maximize statistical power to ensure the accuracy of parameter estimation in subsequent mediation effect tests and reduce the impact of sampling errors on Bootstrap confidence interval calculations (Fritz and MacKinnon, 2007; Wen and Ye, 2014), this study merged these two split samples (*N* = 512) and used the total sample as the final analysis database of the educationlist spirit scale for subsequent structural equation modeling path analysis and mediation effect tests. In Lan's (2025) study, it sought to examine the influence of educationalist spirit scale on the collective efficacy of university teachers, the educationalist spirit scale was developed, comprising also four dimensions: moral literacy, knowledge literacy, ability literacy, and psychological literacy. This structure was subsequently validated, thereby confirming this study scale's overall rationality as well. Therefore, according to the content of the questions, the four factors are named: Factor 1: The Educational Ethos and a Nurturing Spirit (5 items), Factor 2: Innovative Competence and Practical Implementation (4 items), Factor 3: Moral Leadership and Exemplary Demonstration (4 items), and Factor 4: Social Responsibility and Mission Responsibility (3 items).

### 4.2 Correlation analysis

The common method bias test adopted Harman single factor method, and the first factor without rotation explains 28.5% of the variance, which is less than the critical standard of 40%, indicating that the common method bias is not severe. To further assess common method bias beyond the Harman test, we adopted the marker variable technique (Lindell and Whitney, 2001). In the existing questionnaire data, “grade” was selected as the marker variable—this variable theoretically has no direct logical correlation with all the core constructs of this study. Calculate the partial correlation coefficients between the labeled variables and each core variable. The partial correlations between this marker and all core variables ranged from −0.06 to 0.08, none of which reached statistical significance (*p* > 0.05) and all were well below the 0.30 threshold. This result indicates that common method bias did not have a substantial impact on the conclusions of this study. The mean, standard deviation, and correlation coefficient of each variable are shown in Table 1. Principal entrepreneurial leadership, teacher entrepreneurial leadership, teacher efficacy, and educationalist spirit and their various dimensions are significantly positively correlated (*r* values between 0.421 and 0.695, *p* < 0.01). This provides preliminary support for subsequent hypotheses testing. In addition, hypothesis 6 has been verified that there is a significant relationship between teacher entrepreneurial leadership and teacher efficacy of students in normal universities. The square roots of AVE for each construct-−0.804 (principal entrepreneurial leadership), 0.698 (teacher entrepreneurial leadership), 0.785 (teacher efficacy), and 0.815 (educationalist spirit), were all greater than the absolute values of inter-construct correlations (the largest being *r* = 0.695, between principal entrepreneurial leadership and teacher efficacy). The results indicate that there is good discriminant validity between each construct, meaning that the four variables can be clearly distinguished in measurement. To test whether teacher entrepreneurial leadership and perceived principal entrepreneurial leadership are two independent constructs, the study used the (Fornell and Larker, 1981) to conduct discriminant validity tests. The AVE square roots of the two variables were 0.698 and 0.804, respectively, both of which were greater than the correlation coefficient between the two (*r* = 0.421), indicating sufficient discriminant validity between the two constructs (Fornell and Larker, 1981; Wen and Ye, 2014). In this study, students in education were able to clearly distinguish between teacher entrepreneurial leadership and principal entrepreneurial leadership.

**Table 1:** Descriptive statistics and inter-correlation matrix.

Variable	(1)	(2)	(3)	(4)
(1) Principal Entrepreneurial Leadership	**0.804**			
(2) Teacher Entrepreneurial Leadership	0.421^**^	**0.698**		
(3) Teacher Efficacy	0.695^**^	0.463^**^	**0.785**	
(4) Educationalist Spirit	0.549^**^	0.522^**^	0.618^**^	**0.815**
**Mean**	3.695	3.413	3.634	3.672
**SD**	0.881	0.589	0.625	0.871

Bold diagonal values are square roots of AVE (√AVE); off-diagonal values are Pearson correlation coefficients;

^**^p < 0.01.

### 4.3 Hypotheses test

The analysis employing structural equation modeling encompassed several key steps. Firstly, the study established a measurement model to verify whether the latent variable is effectively measured by its observed indicators, which is conducting reliability and validity tests. To evaluate whether the proposed four-factor model was optimal, the study tested three alternative measurement models via CFA in Table 2: (1) a one-factor model, (2) a two-factor model (combining Principal Entrepreneurial Leadership and Teacher Efficacy), and (3) a three-factor model (combining Principal and Teacher Entrepreneurial Leadership). The four-factor hypothesized model demonstrated the best fit (χ^2^/df = 2.03, CFI = 0.96, TLI = 0.95, RMSEA = 0.058, SRMR = 0.039). In contrast, the one-factor model showed poor fit (CFI = 0.72, RMSEA = 0.142), ruling out serious common method bias. The two-factor (CFI = 0.84, RMSEA = 0.098) and three-factor (CFI = 0.88, RMSEA = 0.085) models also fell short of acceptable fit thresholds. The significant deterioration in fit from the four-factor model to all nested alternatives, further confirmed that treating the four constructs as distinct factors was empirically justified (Wen and Ye, 2014).

**Table 2:** Comparison of alternative measurement models.

Model	Factor structure	*χ^2^*/df	CFI	TLI	RMSEA
Model 1	One-factor (all items load onto a single factor)	4.68	0.72	0.68	0.142
Model 2	Two-factor (PEL+TE combined; TEL and ES separate)	3.42	0.84	0.81	0.098
Model 3	Three-factor (PEL+TEL combined; TE and ES separate)	2.96	0.88	0.86	0.085
Model 4	Four-factor (hypothesized model; all constructs separate)	2.03	0.96	0.96	0.058

PEL, Principal Entrepreneurial Leadership; TEL, Teacher Entrepreneurial Leadership; TE, Teacher Efficacy; ES, Educationalist Spirit.

Then, construct a structural model, which were tested via path analysis. It is necessary to check whether there is severe multicollinearity between the predictor variables, which can lead to distortion in path coefficient estimation. Subsequently, explore how this relationship occurs and dissect the effects involved, the bootstrap method was applied to validate the hypothesized relationships among the latent variables. The detailed results of the SEM analysis are presented as follows. Above all, apply structural equation modeling to test for direct and indirect effects. The model fits well: χ^2^/df = 2.38, CFI = 0.95, TLI = 0.94, RMSEA = 0.072. The path analysis results show in Table 3.

**Table 3:** Path relationship verification of the structural model.

Path relationship	Estimate	S. E.	C. R.	*P*
*Model without mediators*				
Principal Entrepreneurial Leadership —> Educationalist Spirit	0.311	0.034	6.197	^***^
*Model with mediators*				
Principal Entrepreneurial Leadership —> Teacher Efficacy	0.483	0.051	9.135	^***^
Principal Entrepreneurial Leadership —> Educationalist Spirit	0.219	0.045	4.982	^***^
Teacher Entrepreneurial Leadership —> Educationalist Spirit	0.194	0.061	3.577	^***^
Teacher Efficacy —> Educationalist Spirit	0.451	0.039	8.861	^***^
Principal Entrepreneurial Leadership —> Teacher Entrepreneurial Leadership	0.096	0.047	2.413	^**^

^**^P < 0.01,

^***^P < 0.001.

**Figure 4:**
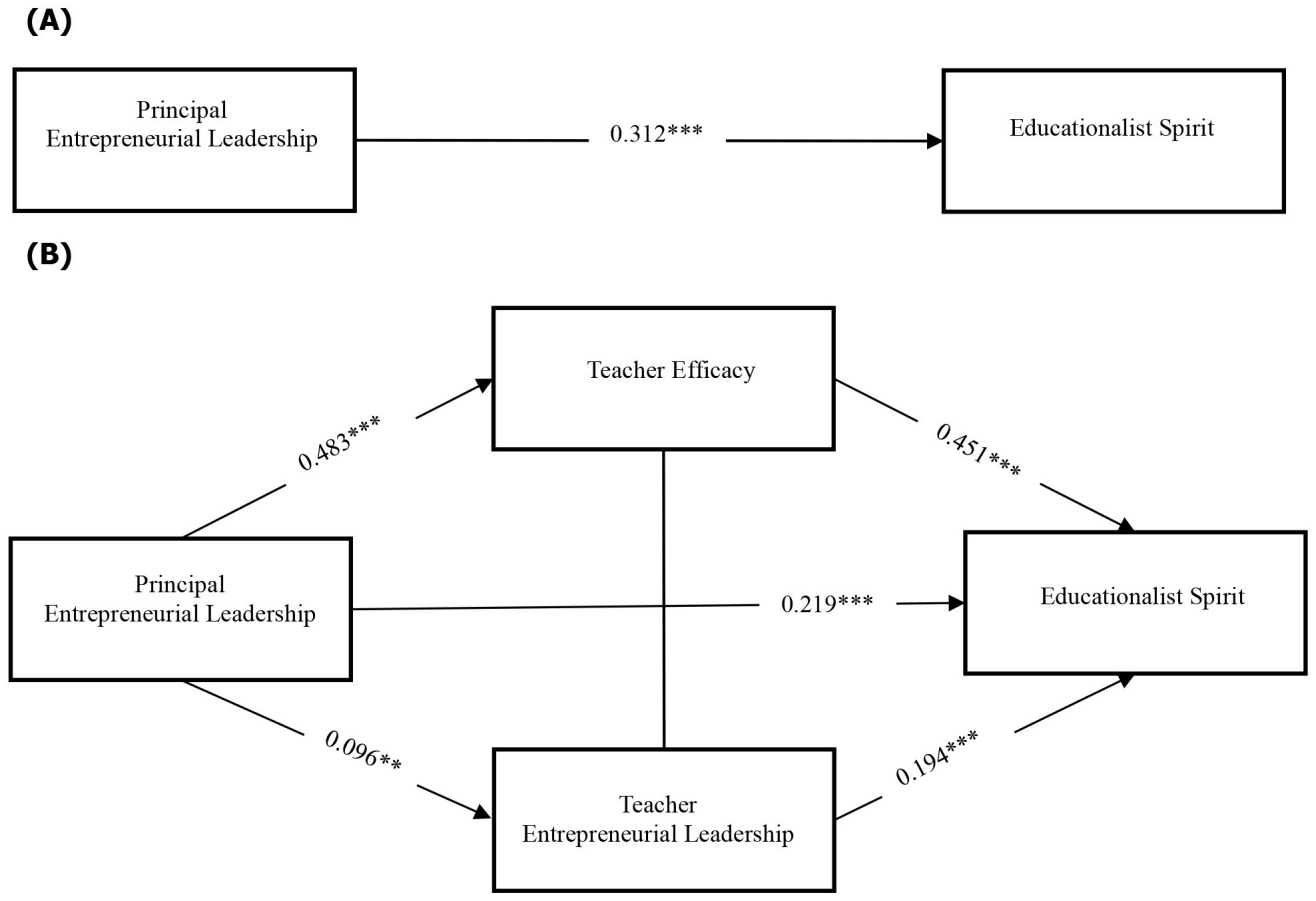
Structural model outcome. **(A)** Direct effect, **(B)** Indirect effect.

Finally, the Bootstrap method was used to examine the mediating effect of teacher efficacy. Bootstrap is important because it no longer relies on strict assumptions of normal distribution. Through repeated sampling techniques, it can more accurately estimate the confidence interval of indirect effects. It only need to check whether this interval contains 0 to directly determine whether the mediating effect is valid (Hou et al., 2004). The results showed that the direct effect of principal entrepreneurial leadership on the educationalist spirit was 0.31, with a 95% confidence interval of [0.21, 0.41]; The indirect effect of teacher efficacy was 0.22, with a 95% confidence interval of [0.15, 0.29], and the intervals do not include 0, indicating significant indirect effects. Due to the significant direct and indirect effects, teacher efficacy partially mediates and H7 is supported. The structural model outcome presented in Figure 4.

## 5 Research summary and discussions

The study produced two primary findings. A key outcome was the development and validation of a educationalist spirit scale. This scale is multidimensional, incorporating four specific factors: the educationalist ethos and nurturing spirit, innovative competence and practical implementation, moral leadership and exemplary demonstration, social responsibility and mission responsibility. This scale possesses reliability and validity, providing a reliable measurement tool for subsequent educationalist spirit research. In addition, the integrated model constructed in this study is supported by data, revealing that entrepreneurial leadership and teacher efficacy are the two key driving forces shaping the educationalist spirit of students in the education profession. Specifically, entrepreneurial leadership demonstrates both direct and indirect effects on the educationalist spirit, with the indirect effect being mediated through the internal psychological mechanism of enhanced teacher efficacy.

Central to the research question, the integrated model received empirical support. It elucidates a dual-pathway mechanism underlying the cultivation of educationalist spirit in normal universities. The direct path from principal entrepreneurial leadership to educationalist spirit (β = 0.31) and teacher entrepreneurial leadership to educationalist spirit (β = 0.45) highlight the significant influence of the proximal institutional environment. When faculty and institutional leaders exhibit entrepreneurial behaviors, such as championing innovation, empowering students, and embracing calculated risks. They establish a role script and foster a supportive climate that directly legitimizes and nurtures corresponding attitudes and aspirations in future teachers. This finding extends the scope of entrepreneurial leadership beyond organizational performance outcomes to the deeper domain of professional identity and ethos formation.

The indirect path, where principal entrepreneurial leadership boosts teacher efficacy (β = 0.48), which in turn strongly predicts educationalist spirit (β = 0.45), offers a deeper, agentic explanation. This pathway aligns perfectly with spiritual leadership theory as well as social cognitive theory. Entrepreneurial leaders act as influential social models and provide mastery experiences (e.g., through innovative project assignments, supportive feedback on teaching attempts). These experiences enhance normal students belief in their own capabilities (efficacy). A strong sense of efficacy is not merely about confidence in executing tasks; it is the foundational belief that one can make a difference. This belief empowers individuals to embrace the challenges inherent in the lofty ideals of educationalist spirit, to persist in innovation despite setbacks, to lead morally with conviction, and to support significant social responsibility. The partial mediation indicates that leadership operates through both shaping the environment and fortifying the individual's psychological core.

Embodying the pursuit of global citizenship and enlightening minds through culture constitutes the elevated perspective that the teaching body must foster, the defining social responsibility of contemporary Chinese educators, and the paramount realm of the educationalist spirit. Bearing the crucial responsibility of moral guidance and education, the teaching must aspire to global citizenship and focus on the future, disseminating common values of humanity (Lei, 2024). This is essential to developing students equipped with a wide international perspective and deep-rooted patriotic devotion. Students are called upon to undertake a deep study of advanced Eastern and Western cultures, assimilating exemplary achievements from all human civilization. The goal is to steer students toward becoming high-quality international talents, equipped with national pride, a worldwide perspective, and creative skills, and prepared to dedicate their youth to the cause of fostering a human community with a shared future.

## 6 Limitations and implications

This study selected Henan Normal University and Zhengzhou Normal University as representatives of normal universities in Henan Province for research. Although this sampling strategy takes into account the two key levels of “top-notch leadership” and “local foundation” in normal education within the province, there are significant differences between the two sample universities in terms of educational positioning, student quality, and resource acquisition. This difference sampling promotes to reveal extreme situations, it may also underestimate the universal characteristics of intermediate level universities. Future research may consider expanding the sample size to include more normal univeristies from different levels and regions, in order to construct a more comprehensive explanatory framework. Besides, this study employed a cross-sectional design, with all data collected at a single time point. Although the hypothesized mediation effects were supported by statistical tests (e.g., bootstrapping), cross-sectional data cannot empirically establish temporal precedence among the variables. Therefore, the indirect effects identified in this study should not be interpreted as causal evidence. The mediation results are best understood as statistical associations that are consistent with the proposed theoretical framework, rather than as deterministic causal pathways. Future research is encouraged to adopt longitudinal or experimental designs to examine the dynamic interplay and causal direction among these constructs by controlling for baseline levels and tracking changes over time.

To underpin economic and social development, teaching and research must be propelled by the educationalist spirit. Universities, serving as the main body of basic research and the source of key technological breakthroughs, undertake the fundamental tasks of talent development and moral education. Hence, championing the educator spirit should be established as a key foundation for achieving high-quality, integrated progress across all initiatives. This calls for a multi-faceted approach: implementing institutional mechanisms at the national level to generate the educationalist spirit; building a supportive cultural ecosystem at the societal level for it to flourish; and forging the foundational intrinsic motivation within individuals to sustain it. It is recommended that school principals follow a progressive process from learning to internalization, and then to externalization and optimization, in order to further shape their own leadership and achieve a deep integration of the educationalist spirit and the leadership of principals. The promotion path of the educationalist spirit is a multidimensional and complex process, which relies on the individual efforts of educators, also requires the joint cooperation and support of educational institutions, various sectors of society, and governments, aiming to provide theoretical guidance and practical paths for the promotion and practice of the educationalist spirit.

## Data Availability

The original contributions presented in the study are included in the article/Supplementary material, further inquiries can be directed to the corresponding author.
