# Supplementary material for: The structure and influencing factors of educationalist spirit among students majoring in education: an integrated model based on entrepreneurial leadership and teacher efficacy
Source: Front Psychol. 2026 Aug 26;17:1834394. doi: 10.3389/fpsyg.2026.1834394 (PMC13553365; doi:10.3389/fpsyg.2026.1834394)
Supplement: Supplementary file 1 [file Supplementary_file_1.docx]

Supplementary Material

1 **Supplementary Appendix**

**Appendix 1. Educationalist spirit items and factor loading**

| **Educationalist spirit (Cronbach’s Alpha: 0.869)** | **Factor loading** |
| --- | --- |
| ***The Educational Ethos and a Nurturing Spirit*** |  |
| I genuinely love the field of education and feel proud of it. | 0.652 |
| Being with students and witnessing their growth process can bring me great happiness. | 0.724 |
| Despite the inherent challenges and routine tasks in teaching, it is precisely this context that reveals a profound sense of purpose, compelling my wholehearted commitment. | 0.708 |
| I firmly believe that education is a great cause that changes individual destiny and promotes social progress. | 0.673 |
| I am full of aspirations and expectations for my future career as a teacher. | 0.651 |
| ***Innovative Competence and Practical Implementation*** |  |
| I am willing to pay attention to and learn advanced educational concepts and teaching methods both domestically and internationally. | 0.764 |
| I am dedicated to incorporating advanced teaching technologies into my pedagogy with the goal of significantly elevating teaching efficacy. | 0.729 |
| I maintain that teachers must be prepared to tailor their pedagogical approaches in response to the individual characteristics and dynamic needs of each student. | 0.696 |
| I hope to develop a unique teaching style through my own exploration and practice. | 0.652 |
| ***Moral Leadership and Exemplary Demonstration*** |  |
| It is a fundamental conviction that educators are obligated to exemplify ethical conduct and serve as moral compasses for their students. | 0.755 |
| I am committed to safeguarding the positive image of the teaching profession through my behaviors and conscious conduct. | 0.703 |
| I endorse the view that ‘profound knowledge serves as the teacher, and upright conduct sets the example’ constitutes a fundamental requirement for the teaching profession. | 0.697 |
| I proactively seek to positively influence the students around me through the deliberate integration of positive values. | 0.682 |
| ***Social Responsibility and Mission Responsibility*** |  |
| I regard the cultivation of exceptional students as my premier contribution to the future of my nation and society. | 0.712 |
| I deeply feel the great responsibility of nurturing future pillars for the country. | 0.685 |
| I am keenly aware of the profound responsibility inherent in nurturing the individuals who will become the future pillars of the nation. | 0.694 |

**Appendix 2. Principal entrepreneurial leadership items and factor loading**

| **Principal Entrepreneurial Leadership (Cronbach’s Alpha: 0.819)** | **Factor loading** |
| --- | --- |
| ***General Entrepreneurial Leadership*** |  |
| Spends time on new business development. | 0.671 |
| Points out the competition’s weaknesses and how we could take advantage of them. | 0.665 |
| Listens to and acts upon students complaints. | 0.698 |
| Challenges us to think about new and better ways to do our work. | 0.704 |
| Assertively communicates to upper manager regarding how things could be run better. | 0.659 |
| Looks for creative ways to manage, use, or rearrange company assets and resources. | 0.726 |
| Passionately looks for new ways to grow the business. | 0.713 |
| Motivates us to think of innovative ways to beat the competition. | 0.686 |
| ***Explorer Behavior*** |  |
| Effectively pitch new education ideas to upper management. | 0.726 |
| Supports our suggestions for improving the organizations. | 0.695 |
| Communicates a vision of how the organization could be better in the future if we were to make certain improvements. | 0.690 |
| Encourages us to challenge the status quo. | 0.673 |
| Prioritizes customers needs when making changes to our organization. | 0.651 |
| Tells us where we stand in regards to the competition. | 0.663 |
| Encourages us to innovate in how we do our work. | 0.724 |
| Actively identifies, develops, and goes after new business opportunities. | 0.658 |
| Makes sure that we have the right team of people in place to successfully capture these new opportunities. | 0.721 |
| ***Miner Behavior*** |  |
| Displays enthusiasm for us learning new skills. | 0.688 |
| Quickly takes a different direction when results aren’t being achieved. | 0.604 |
| Encourages others to take the initiative and action for their own ideas. | 0.741 |
| Motivates people to think about how to do their work in new and interesting ways | 0.707 |
| Allots time to helping others find ways to improve our products and services. | 0.698 |
| Creates a climate that encourages continuous service improvement. | 0.701 |
| ***Accelerator Behavior*** |  |
| Willingly moves ahead with a promising new approach when others might hold back. | 0.688 |
| Promotes an environment where risk taking is encouraged. | 0.645 |
| Identifies, encourages, and protects rebels who might think and act differently than the majority of employees. | 0.618 |
| Encourages others to outwit and outmaneuver the organization’s bureaucracy | 0.664 |
| Quickly utilizes different approaches to overcoming obstacles when the initial one doesn’t work. | 0.713 |
| Creates an environment where people feel free to try new things. | 0.689 |
| Challenges us to creatively discover ways to do more with less. | 0.731 |
| Demonstrates an entrepreneurial orientation at work. | 0.688 |
| Pushes the organization to be fast, flexible, and adaptable so that we can react quickly when new business opportunities arise. | 0.721 |
| Actively fights the encroachment of bureaucracy in the organizations. | 0.652 |
| Utilizes an extensive network of people throughout the organization that is willing to help if called upon. | 0.695 |
| ***Integrator Behavior*** |  |
| Analyzes work flow, resources, and procedures to see how we can do our work better and faster with better impact for the customer. | 0.686 |
| Expects us to constructively identify and solve cross-organizational problems and issues. | 0.724 |
| Willingly listens to suggestions from others about how to do things differently. | 0.706 |
| Support us in fighting for changes which will improve the way the organization works. | 0.711 |
| Strives to build an innovate culture within our organization. | 0.723 |
| Encourages entrepreneurial thinking and risk taking. | 0.741 |
| Reacts quickly to remove organizational barriers that get in the way of doing business. | 0.698 |
| Encourages open communications and ideas sharing across organizational unites and functions. | 0.727 |
| Keeps the organization informed and updated on industry trends and competitor strategies. | 0.721 |
| Actively encourages business improvement suggestions throughout the organization. | 0.714 |
| Takes action to implement agreed suggestions. | 0.679 |
| Keeps the organization focused on its core strategy but also supports new business initiatives. | 0.701 |
| Puts aside money outside of the normal budget process in order to fund and support innovate ideas. | 0.645 |
| Encourage employees to challenge their decisions. | 0.663 |

**Appendix 3. Teacher entrepreneurial leadership items and factor loading**

| **Teacher Entrepreneurial Leadership (Cronbach’s Alpha: 0.675)** | **Factor loading** |
| --- | --- |
| ***General Entrepreneurial Leadership*** |  |
| Spends time on new education development. | 0.658 |
| Points out the competition’s weaknesses and how we could take advantage of them. | 0.661 |
| Listens to and acts upon students complaints. | 0.681 |
| Challenges us to think about new and better ways to do our work. | 0.659 |
| Assertively communicates to upper manager regarding how things could be run better. | 0.677 |
| Looks for creative ways to manage, use, or rearrange company assets and resources. | 0.728 |
| Passionately looks for new ways to grow the organization. | 0.745 |
| Motivates us to think of innovative ways to beat the competition. | 0.673 |
| ***Explorer Behavior*** |  |
| Effectively pitch new education ideas to upper management. | 0.686 |
| Supports our suggestions for improving the organizations. | 0.671 |
| Communicates a vision of how the organization could be better in the future if we were to make certain improvements. | 0.663 |
| Encourages us to challenge the status quo. | 0.674 |
| Prioritizes customers needs when making changes to our organization. | 0.638 |
| Tells us where we stand in regards to the competition. | 0.611 |
| Encourages us to innovate in how we do our work. | 0.771 |
| Actively identifies, develops, and goes after new business opportunities. | 0.721 |
| Makes sure that we have the right team of people in place to successfully capture these new opportunities. | 0.708 |
| ***Miner Behavior*** |  |
| Displays enthusiasm for us learning new skills. | 0.731 |
| Quickly takes a different direction when results aren’t being achieved. | 0.609 |
| Encourages others to take the initiative and action for their own ideas. | 0.715 |
| Motivates people to think about how to do their work in new and interesting ways | 0.743 |
| Allots time to helping others find ways to improve our products and services. | 0.684 |
| Creates a climate that encourages continuous service improvement. | 0.715 |
| ***Accelerator Behavior*** |  |
| Willingly moves ahead with a promising new approach when others might hold back. | 0.685 |
| Promotes an environment where risk taking is encouraged. | 0.613 |
| Identifies, encourages, and protects rebels who might think and act differently than the majority of employees. | 0.627 |
| Encourages others to outwit and outmaneuver the organization’s bureaucracy | 0.658 |
| Quickly utilizes different approaches to overcoming obstacles when the initial one doesn’t work. | 0.691 |
| Creates an environment where people feel free to try new things. | 0.723 |
| Challenges us to creatively discover ways to do more with less. | 0.708 |
| Demonstrates an entrepreneurial orientation at work. | 0.696 |
| Pushes the organization to be fast, flexible, and adaptable so that we can react quickly when new organization opportunities arise. | 0.772 |
| Actively fights the encroachment of bureaucracy in the organization. | 0.653 |
| Utilizes an extensive network of people throughout the organization that is willing to help if called upon. | 0.714 |
| ***Integrator Behavior*** |  |
| Analyzes work flow, resources, and procedures to see how we can do our work better and faster with better impact for the student. | 0.672 |
| Expects us to constructively identify and solve cross-organizational problems and issues. | 0.731 |
| Willingly listens to suggestions from others about how to do things differently. | 0.718 |
| Support us in fighting for changes which will improve the way the organization works | 0.664 |
| Strives to build an innovate culture within our organization. | 0.695 |
| Encourages entrepreneurial thinking and risk taking. | 0.729 |
| Reacts quickly to remove organizational barriers that get in the way of doing business. | 0.654 |
| Encourages open communications and ideas sharing across organizational unites and functions. | 0.697 |
| Keeps the organization informed and updated on industry trends and competitor strategies. | 0.702 |
| Actively encourages business improvement suggestions throughout the organization. | 0.721 |
| Takes action to implement agreed suggestions. | 0.693 |
| Keeps the organization focused on its core strategy but also supports new business initiatives. | 0.697 |
| Puts aside money outside of the normal budget process in order to fund and support innovate ideas. | 0.651 |
| Encourage employees to challenge their decisions. | 0.594 |

**Appendix 4. Teacher efficacy items and factor loading**

| **Teacher Efficacy (Cronbach’s Alpha: 0.921)** | **Factor loading** |
| --- | --- |
| ***Student Engagement*** |  |
| How much can you do to get through to the most difficult students? | 0.476 |
| How much can you do to help your students think critically? | 0.485 |
| How much can you do to get students to believe they can do well in  school assignment? | 0.503 |
| How much can you do to foster student creativity? | 0.451 |
| How well can you respond to difficult questions from your students? | 0.489 |
| How much can you do to adjust your lessons based on individual students’ competency? | 0.443 |
| To what extent can you provide additional information explanation or example when students are confused? | 0.428 |
| ***Instructional Practice*** |  |
| How much can you do to control disruptive behavior in the classroom? | 0.514 |
| To what extent can you define your expectations for student behavior? | 0.465 |
| How much can you do to get student to follow classroom rules? | 0.542 |
| How much can you do to help students who have failed in learning to improve their understanding? | 0.530 |
| How well can you respond to changing/misdemeanor students? | 0.472 |
| ***Classroom Management*** |  |
| How much can you do to motivate students who show low interest in school assignment? | 0.583 |
| How much can you do to help your students value learning? | 0.514 |
| Communicates a vision of how the organization could be better in the future if we were to make certain improvements. | 0.536 |
| How much can you assist families in helping their children do well in school? | 0.547 |
| How well can you implement alternative strategies in your classroom? | 0.498 |
| How well can you provide appropriate challenges for high performance students? | 0.516 |
